# The Interplay of Cohesin and the Replisome at Processive and Stressed DNA Replication Forks

**DOI:** 10.3390/cells10123455

**Published:** 2021-12-08

**Authors:** Janne J. M. van Schie, Job de Lange

**Affiliations:** Cancer Center Amsterdam, Department of Human Genetics, Section Oncogenetics, Amsterdam University Medical Centers, De Boelelaan 1118, 1081 HV Amsterdam, The Netherlands

**Keywords:** sister chromatid cohesion, DNA replication, replisome, cohesin complex, DNA entrapment, cohesion establishment, SMC3 acetylation, DNA replication stress, fork stalling

## Abstract

The cohesin complex facilitates faithful chromosome segregation by pairing the sister chromatids after DNA replication until mitosis. In addition, cohesin contributes to proficient and error-free DNA replication. Replisome progression and establishment of sister chromatid cohesion are intimately intertwined processes. Here, we review how the key factors in DNA replication and cohesion establishment cooperate in unperturbed conditions and during DNA replication stress. We discuss the detailed molecular mechanisms of cohesin recruitment and the entrapment of replicated sister chromatids at the replisome, the subsequent stabilization of sister chromatid cohesion via SMC3 acetylation, as well as the role and regulation of cohesin in the response to DNA replication stress.

## 1. Introduction

Every cell division requires the duplication and separation of the entire genome to ensure that each daughter cell receives a complete copy. A critical step for faithful chromosome segregation is the stable cohesion between replicated sister chromatids, mediated by the cohesin complex and established at the DNA replication fork. The cohesin complex consists of two coiled-coil subunits, SMC1A and SMC3, the kleisin subunit RAD21, and the additional subunits STAG1/2 and PDS5A/B. Together, these components form a ring-shaped structure, which can engage DNA in topological and non-topological manners and is essential for proliferation [1,2]. From late telophase onwards, the NIPBL/MAU2 loader complex promotes cohesin loading onto chromatin at accessible, presumably nucleosome-free regions [3,4,5,6]. Cohesin can translocate from initial loading sites [7,8,9] until it reaches physical barriers on chromatin [7,10,11,12]. Before DNA replication, the association of cohesin with chromatin is dynamic due to the release activity of WAPL [13,14]. During DNA replication, two changes occur to establish sister chromatid cohesion. First, cohesin topologically co-entraps two sister chromatids. Second, cohesion is stabilized by acetylation of the SMC3 subunit by the acetyltransferase ESCO2 to render cohesin resistant to WAPL activity [15]. Consequently, the residence time of a subpool of cohesin on chromatin increases from approximately 20 min in G1 to multiple hours in G2 [16,17]. In mitosis, cohesin rings are removed in two steps: A phosphorylation-dependent process triggers WAPL-mediated cohesin release from chromosome arms during prophase, followed by protease-dependent cleavage of remaining rings at centromeres at anaphase onset, which triggers the separation of sister chromatids to opposite sides of the cell [1]. By connecting different DNA segments, cohesin has functions in genome organization, transcriptional regulation, and post-replicative double strand break (DSB) repair. Several excellent reviews covering these topics are published elsewhere [18,19,20]. Here, we review the complex interplay of cohesin and DNA replication. Table 1 presents the yeast and human names of relevant cohesion and replication factors that are discussed. We describe how the cohesin complex is recruited to the replisome, how many replisome factors play dual roles in cohesion establishment and DNA replication and to what extent these processes can be separated, as well as the role of cohesin and cohesion-associated factors in the response to DNA replication stress.

## 2. Replisome Assembly and DNA Replication

**Figure 1:**
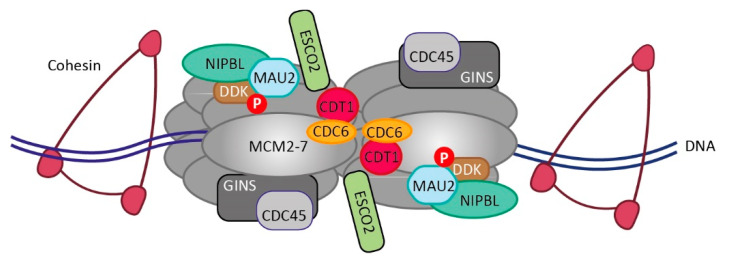
The pre-RC complex interacts with multiple cohesion factors. Origins of replication on DNA are marked by multi-subunit ORC complexes (not shown), which, in concert with CDT6 and CDT1, load MCM2-7 complexes onto DNA as double hexamers. DDK-dependent phosphorylation promotes binding of the cohesin loader complex NIPBL-MAU2 and the cohesin complex to pre-RCs. Recruitment of the acetyltransferase ESCO2 can occur prior to DNA replication via an interaction with MCM2-7. In S-phase, phosphorylation of MCM further stimulates CMG assembly (CDC45-MCM-GINS) and subsequent DNA replication. For further explanation, see text.

Starting from late mitosis to early G1, at sites marked with the origin recognition complex (ORC), two hexameric mini-chromosome maintenance 2-7 (MCM2-7) helicase complexes are loaded in concert with CDC6 and CDT1 to form a pre-Replication Complex (pre-RC) (Figure 1) [21,22]. Its activation in S phase depends on Dbf4-dependent kinase (DDK) and Cyclin-dependent kinases (CDKs) [23], which phosphorylate multiple residues on the MCM complex. This facilitates the binding of additional replication factors, including the tetrameric GINS complex and CDC45, to form the replicative DNA helicase complex CDC45/MCM2-7/GINS (CMG). The active CMG encircles a single strand of DNA and translocates the leading strand template with 3′–5′ directionality, thereby separating the leading from the lagging strand, to provide a template for DNA synthesis [24] and forming the basis for further replisome assembly [25,26].

**Figure 2:**
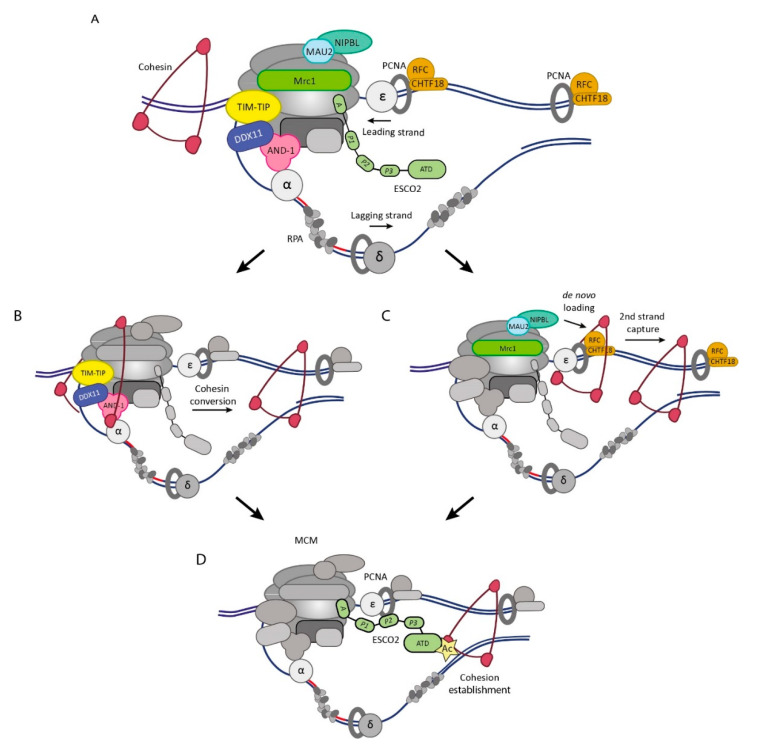
Entrapment of sister chromatids during DNA replication: (**A**) Upon separation of the two parental DNA strands by the CMG, the DNA polymerases δ and ε synthesize DNA assisted by PCNA. Cohesion establishment factors are indicated in color. (**B**) The conversion pathway consists of TIMELESS-TIPIN, DDX11, and AND-1, which facilitate the transfer of pre-loaded cohesin rings to tether the two sister chromatids behind the fork, in a lagging strand oriented process. (**C**) The de novo pathway, consisting of the PCNA loader CHTF18-RFC, Mrc1 (established in yeast) and NIPBL-MAU2, may involve initial capture of the leading strand, followed by lagging strand capture while it is still single stranded. (**D**) ESCO2 acetylates SMC3 to establish cohesion. ESCO2 interacts with the replisome through PCNA interacting domains (depicted as P1–P3) and the MCM-interacting Box A domain (**A**). ATD, acetyltransferase domain. For further details, see text.

DNA replication is carried out by the concerted action of DNA polymerases α, δ, and ε (POLα, POLδ, and POLε). The POLα–Primase complex creates RNA primer templates which are extended by POLδ on the lagging strand and by POLε on the leading strand (Figure 2A). POLε interacts with MCM to capture the leading strand template for DNA synthesis [27,28] and with PCNA (proliferating cell nuclear antigen) on its opposite side, from where the nascent daughter DNA exits [29]. PCNA is a homotrimeric DNA sliding clamp that encircles DNA and stimulates the processivity of DNA polymerases but also acts as a docking platform to recruit additional proteins to the replisome via PCNA interacting protein (PIP) boxes [30]. At the lagging strand, CMG interacts with AND-1 (Ctf4 in yeast) [31,32]. Ctf4 homotrimers function as a hub in the replisome, connecting the CMG complex and POLα-Primase in the proximity of the lagging strand [31,33]. PCNA on the lagging strand interacts with POLδ to mediate lagging strand elongation [34]. Furthermore, POLδ gradually displaces the RNA primers to allow their removal by the PCNA-bound Flap Endonuclease 1 (FEN1) and the DNA2 nuclease [34,35,36]. DNA ligase 1 (LIG1) connects the nicked ends to phosphate backbones to fully mature the Okazaki fragments.

Replication complexes can function as chromatin acceptor sites for cohesin loading. Cohesin and NIPBL/MAU2 (Scc2/Scc4 in yeast) interact with MCM2-7 in multiple cellular systems, an interaction stimulated by DDK (Figure 1) [37,38,39,40,41]. In Xenopus egg extracts, pre-RC-dependent cohesin loading was shown to take place before origin unwinding and the initiation of DNA replication [40,41]. However, the depletion of Cdc6 in budding yeast [42] and Cdt1 in Drosophila [43] revealed that chromatin association of cohesin can occur independently from pre-RC formation and pre-RCs do not seem to be the main cohesin acceptor sites in human cells during telophase and G1 [37,38]. Instead, pre-RCs may function to pre-assemble the cohesin loading machinery to be primed for cohesin loading during active DNA replication. Indeed, the interaction between MCM and NIPBL/MAU2 persists during DNA replication and is necessary for normal chromatin-bound cohesin levels in S/G2 and proper sister chromatid cohesion [37]. Similarly, ESCO2 is recruited to MCM prior to DNA replication (Figure 1) but only becomes active during replication [44,45]. It has been proposed that cohesin organizes replication factories by chromatin looping [38]. Cohesin colocalizes with MCM on chromatin [37,46] and is enriched at sites of active DNA replication [47]. Part of the observed accumulation of cohesin at pre-RC sites [43] may be a consequence of hampered cohesin translocation imposed by chromatin-bound MCM [12].

### Nucleosomes and Chromatin Remodeling

Local chromatin environment influences cohesion establishment. Nucleosomes inhibit in vitro cohesin loading, and nucleosome-free regions are necessary for efficient cohesin loading [3]. Chromatin remodelers from the SWI/SNF family slide or evict nucleosomes in an ATP-dependent manner, thereby rendering the DNA accessible to loading of DNA interacting factors, including DNA repair and transcription factors [48]. In yeast, the SWI/SNF family member ‘remodeling the structure of chromatin’ (RSC) contributes to sister chromatid cohesion [49,50]. The RSC complex locally depletes the chromatin of nucleosomes to create a template for cohesin loading and concomitantly acts as a chromatin acceptor of the cohesin loading complex by a direct physical interaction [3,6]. Loss of the human RSC orthologue BAF, frequently mutated in the BAF180 subunit in cancer, results in sister chromatid cohesion defects and aneuploidy [51]. The histone acetyltransferase bromodomain-containing protein 4 (BRD4), implicated in nucleosome eviction [52], can interact with NIPBL, and its mutation underlies a syndrome that resembles the cohesinopathy Cornelia de Lange Syndrome [53]. Furthermore, the human ISWI (SNF2/SMARCA5)-containing chromatin remodeling complex interacts with cohesin and promotes cohesin loading [4], and yeast Chd1 (Chromodomain Helicase DNA Binding Protein 1) is involved in sister chromatid cohesion [54]. Some of these remodelers travel with the replication fork, where extensive chromatin remodeling takes place to maintain processive DNA replication [55,56,57]. Chromatin remodeling is necessary for cohesin loading at stalled replication forks [58] and cohesion establishment is coupled to replication-coupled nucleosome assembly [59]. Nevertheless, the exact contribution of chromatin remodelers in cohesion establishment directly at unchallenged replication forks remains poorly understood.

## 3. Sister DNA Entrapment at Processive Replication Forks

In principle, sister chromatid cohesion can be established via either pre-loaded cohesin that remains associated during replication fork passage or de novo cohesin loading behind the fork. Considering that the replisome size is about 20 nm [27] and the cohesin diameter may be up to ~40 nm [60,61], the replisome theoretically may pass through cohesin rings, leading to passive entrapment. However, while cohesin can move past nucleosomes (~10 nm) in vitro, larger replisome-sized DNA-bound proteins of ~20 nm form an impenetrable barrier [10,11], and sub-20 nm sized DNA translocases, including RNA polymerases, translocate cohesin instead of passing through the cohesin lumen in vitro [10,11]. Note that this is different during loop extrusion, which may be non-topological and is largely unimpeded even by 200 nm barriers [62]. Nevertheless, in *S. cerevisiae*, cohesion establishment can take place in the absence of Scc2-dependent cohesin loading during S phase [63,64], and in U2OS cells with inactivated WAPL-mediated cohesin turnover, pre-loaded cohesin can remain associated with chromatin during replication fork passage [65]. Together, this indicates that pre-loaded cohesin can generate sister chromatid cohesion but requires (co-factors for) transient opening or structural remodeling of the ring.

These observations do not exclude that de novo loading also contributes to cohesion establishment. Indeed, it was shown that Scc2 activity during S phase is still required to build efficient sister chromatid cohesion [66,67]. This could be facilitated by the presence of NIPBL-MAU2 in the vicinity of active replication forks through its interaction with MCM during DNA replication [37], although other methods of NIPBL-MAU2 recruitment to the replication fork may exist. In line, two epistasis groups of replication-associated cohesion factors have been identified, which indicates the existence of parallel cohesion establishment pathways [68,69]. In yeast, cells with defects in one cohesion establishment pathway are viable, but the combined loss of both pathways leads to synthetic lethality due to the complete loss of cohesion [68,69], a genetic interaction seemingly conserved in vertebrates [70]. Recent evidence using cell cycle-specific expression of yeast *Scc2* mutant alleles indicates that these epistasis groups are in line with the (loading-independent) conversion and (loading-dependent) de novo pathway [71] (illustrated in Figure 2). An overview of these factors is presented in Table 1 and further discussed below.

### 3.1. Conversion Pathway

The components of epistasis group 1 (TIMELESS-TIPIN, AND-1 and DDX11) all play multiple roles in replication fork dynamics (see Box 1) and additionally promote sister chromatid cohesion, presumably by facilitating the transfer of pre-loaded cohesin to the back of the replisome and convert it into cohesive cohesin (Figure 2B). The Fork Protection Complex (FPC) components TIMELESS and TIPIN (Tof1-Csm3 in yeast) can interact with DNA and RPA [72,73,74,75,76] and with the front of MCM, allowing ideal positioning ahead of the replication fork to sense upcoming DNA obstacles [74,77,78]. During S phase, the FPC promotes the chromatin association of cohesin [37,79], as well as cohesion and SMC3 acetylation [68,79,80]. Human TIMELESS interacts with the iron–sulfur cluster 5′ to 3′ DNA helicase DDX11 (Chl1 in yeast) [79]. This interaction is critical for sister chromatid cohesion and depends on a TIMELESS interaction motif, which is conserved in vertebrates but not in budding yeast [81]. In concert with TIMELESS-TIPIN, DDX11/Chl1 engages cohesin during DNA replication and promotes the association of cohesin with chromatin to facilitate sister chromatid cohesion [81,82]. There is some controversy as to whether the helicase activity of DDX11 is important for cohesion establishment. Helicase-dead mutants were found to rescue cohesion defects in *Chl1*Δ yeast [82] but not in *DDX11* deficient avian DT40 cells [83] and human cells [84]. While it remains to be tested whether Chl1 interacts with the FPC in budding yeast, Chl1 can be recruited to the replisome by interacting with Ctf4 (Samora et al., 2016). The Ctf4–Chl1 interaction is mediated by a Ctf4-interacting peptide (CIP) and is critical for sister chromatid cohesion [82]. Although the CIP box is not conserved in vertebrate DDX11 [81], AND-1 binding motifs may be different from yeast CIP boxes [33], and a weak interaction between AND-1 and DDX11 has been reported [85,86]. Thus, while there may be differences in the exact contributing protein–protein interactions, DDX11/Chl1 replisome recruitment is critical for sister chromatid cohesion. DDX11 also interacts with FEN1 and POLδ, suggesting lagging strand localization [85,86]. Since *POL*α or *Primase* mutants in budding yeast cause sister chromatid cohesion defects via a Ctf4/Chl1 dependent pathway [87], cohesion establishment via this epistasis group appears to be coupled to efficient lagging strand synthesis.

### 3.2. De Novo Pathway

Epistasis group 2 contains all three specific subunits of CHTF18-DSCC1-CHTF8-RFC (CHTF18-RFC, Ctf18-RFC in yeast), which is one of the Replication Factor C (RFC) complexes that load and unload the PCNA sliding clamp (see also Box 1). The RFC core components RFC2-5 are complemented with either CHTF18, RFC1, or ATAD5 (Elg1 in yeast), which determine its specific functionality [88]. Ctf18-RFC has two extra subunits (Dcc1 and Ctf8) and loads PCNA at replication forks with a bias for the leading strand [89]. Loss of Ctf18, Dcc1, or Ctf8 all cause cohesion defects and decreased levels of SMC3 acetylation [63,68,89,90,91,92,93]. Rfc1-RFC loads PCNA with a preference for the lagging strand and, although essential for DNA replication, it is dispensable for sister chromatid cohesion [89]. Elg1-RFC unloads PCNA from lagging strands after Okazaki fragment maturation [94,95,96]. The concerted action of these RFCs results in an even distribution of PCNA levels on the leading and lagging strand [89], which seems surprising as the lagging strand is expected to require more PCNA molecules to elongate Okazaki fragments at 150 bp intervals. Possibly, the excess of PCNA on leading strands contributes to the role of Ctf18-RFC in sister chromatid cohesion. Indeed, decreased SMC3 acetylation and sister chromatid cohesion resulting from Ctf18-RFC deletion (and subsequent lower PCNA levels on the leading strand) are rescued by artificially increasing PCNA levels on the lagging strand [89]. Since PCNA interacts with ESCO2/Eco1 to facilitate SMC3 acetylation (a transaction further discussed in Section 3), Ctf18-RFC-loaded PCNA may contribute to ESCO2/Eco1 recruitment. Notably, ESCO2/Eco1 loss is synthetically lethal with the depletion of DDX11, TIMELESS, and TIPIN [68,83,97] but not with Mrc1 or Ctf18-RFC depletion [68,70], suggestive of a shared pathway of Ctf18-RFC and ESCO2/Eco1 in cohesion establishment. However, all cohesin loaded during DNA replication needs to be acetylated to contribute to sister chromatid cohesion, and it remains unclear why one entrapment pathway would be particularly sensitive to reduced SMC3 acetylation. It has been suggested that the reduced SMC3 acetylation upon CTF18 loss reflects a reduction in the total chromatin-bound cohesin, and since the overexpression of ESCO1 or ESCO2 does not rescue the CTF18-RFC-associated defects [70], Ctf18-RFC may not function directly in SMC3 acetylation but instead may promote chromatin association of cohesin. In line with this idea, the de novo pathway as unraveled in yeast requires both Ctf18-RFC and Scc2 during DNA replication [71]. How Ctf18-RFC-dependent PCNA loading would contribute to de novo cohesin loading remains to be investigated.

The fourth component in Epistasis group 2 is Mrc1, which contacts both the front and the back of the replisome [68,69,77,98]. Surprisingly, Mrc1 interacts with the epistasis group 2 members Tof1-Csm3, with which it shares functions in the DNA replication checkpoint in Rad53-Mec1 activation [69,75,99,100,101]. Mrc1 and its presumed vertebrate orthologue CLASPIN both contribute to normal replication fork speed and fork restart after stalling, functions that can be uncoupled from its role in the DNA replication checkpoint response [100,101,102]. Mrc1 loss causes defective cohesion and SMC3 acetylation in budding yeast [68,69,98]. No evidence was found for cohesion defects in CLASPIN-depleted Xenopus egg extracts [103], but human CLASPIN may have a modest contribution to sister chromatid cohesion [104]. How Mrc1 would contribute to sister chromatid cohesion establishment, how this function is related to its epistasis partner Ctf18-RFC and to what extent this is conserved in vertebrates remains to be determined.

De novo loading of cohesin in the wake of the replication fork may establish sister chromatid cohesion in either a single- or two-step chromatid capture process. Cohesin loaded onto DNA in vitro can capture a second DNA molecule promoted by Scc2, which is single stranded but cannot capture a second dsDNA substrate when it already encompasses a first [66,105]. A potential model would be that cohesin is loaded on the leading strand after DNA synthesis by POLε (Figure 2C). Next, the lagging strand is captured while it is still ssDNA, followed by Okazaki fragment synthesis within the cohesin lumen. In line with this model, the DNA synthesis of the complementary DNA strand after ssDNA capture stabilizes the interaction [66]. However, cohesin can also capture two dsDNA molecules simultaneously in vitro [105], suggesting that one-step sister chromatid entrapment might also occur in vivo.

Box 1 **Dual roles of cohesion associated factors in the replication stress response.**

Multiple replisome-associated cohesion factors also contribute to unperturbed DNA replication and the response to DNA replication stress. Here, we briefly discuss to what extent these roles can be uncoupled from their functions in sister chromatid cohesion.

*Ctf18-RFC*—Ctf18-RFC interacts with the leading strand polymerase POLε via Dcc1 and Ctf18, an interaction that contributes to its leading strand recruitment. This interaction is not the only means of leading strand recruitment and is largely dispensable for hydroxyurea (HU) resistance and sister chromatid cohesion, although disruption of the binding to POLε causes defects in the DNA damage response [93,106, 107,108]. Dcc1 can directly bind ssDNA and dsDNA via three conserved Winged Helix (WH) domains, thereby potentially contributing to Ctf18-RFC recruitment [108,109]. Deletion of all three WH domains impairs cohesion and the replication checkpoint similarly to complete Dcc1 deletion, suggesting that DNA binding is crucial for Ctf18-RFC function [109]. Loss of Ctf18-RFC enhances sensitivity to various DNA damage and replication stress inducing drugs and impairs the activation of the DNA replication checkpoint [110,111,112]. In addition, Ctf18-RFC loss causes spontaneous DNA damage and replication fork slowing [70,91,113]. Ctf18-RFC can be recruited to DNA damage sites in non-S-phase cells; it is involved in post-replication DSB repair independent of cohesin recruitment and recruits POLε for post-excision repair gap filling following nucleotide excision repair [114,115]. In the DNA replication checkpoint, Ctf18-RFC is essential to activate Rad53 (yeast orthologue of CHK1) downstream of Mec1 (ATR) to stabilize stalled forks and repress late origin firing [106,111,116,117]. Loss of Elg1 in Ctf18-depleted cells rescues the cohesion defects but aggravates the HU sensitivity and further compromises Rad53 activation [89,96], suggesting that the cohesion function of Ctf18-RFC is uncoupled from replication checkpoint activation. In addition, DNA replication checkpoint activation by Ctf18-RFC does not depend on sister chromatid cohesion, since it is not affected by depletion of Eco1 or cohesin [111]. Furthermore, the replication checkpoint is more sensitive to decreased (chromatin-bound) levels of Ctf18-RFC than cohesion establishment [89,109]. Thus, although the exact nature of these differences remains unclear, functions of Ctf18-RFC in cohesion and DNA replication checkpoint activation seem to be at least partially separated.

*Ctf4/AND-1*—Ctf4 contains multiple functional domains including an N-terminal WD40 repeat domain and the highly conserved SepB domain, which engages in protein–protein interactions via CIP boxes [31,118]. Vertebrate AND-1 has an additional HMG domain involved in DNA binding [33,119], which contributes to replisome recruitment and replication fork speed [120]. The WD40 domain is not required for fork progression but is critical to prevent excessive MRE11-dependent fork resection and ssDNA accumulation and is essential for proliferation [120]. Ctf4 can interact with multiple proteins including nuclease/helicase Dna2 (involved in Okazaki fragment maturation and in end resection in HR), Tof2 (rDNA maintenance), Dpb2 (recruits POLε to the replisome) and Chl1 [82,118,121], thereby assisting in multiple transactions at the replication fork. Ctf4/AND-1 is necessary for normal replication fork speed and S phase progression [82,120,122,123,124], facilitates activation of the DNA replication checkpoint and functions in HR (template switching) and G2/M DSB repair [87,124]. Its cohesion function seems to depend on the recruitment of Chl1 and POLα-Primase in yeast [82,87]. The DNA repair defects caused by mutant POLα–Primase are not a result of defective cohesion [87].

*Tof1-Csm3/TIMELESS-TIPIN*—Tof1-Csm3 is necessary to maintain replication fork speed, activate the DNA replication checkpoint, regulate replication fork restart, and prevent CMG from uncoupling from the polymerases [99,102,125,126]. In addition, Tof1-Csm3 is required for replisome recruitment of Topoisomerase I (Top1) and efficient fork pausing at protein–DNA barriers [127], functions that can be uncoupled from the role of Tof1 in DNA replication checkpoint signaling [77,127,128,129]. TIMELESS has a DNA-binding domain with specificity towards G-quadruplexes (G4s) and facilitates processive DNA replication through regions with G4 structures [130]. In addition, TIMELESS has a PARP1-binding domain, and PARP1-dependent recruitment to DNA damage sites assists HR in S phase and non-S phase cells [131]. To what extent the cohesion function of TIMELESS–TIPIN can be uncoupled from its function in DNA replication remains to be examined.

*Chl1/DDX11*—Mutations in DDX11 underlie the cohesinopathy Warsaw breakage syndrome [132,133]. *DDX11* deficiency sensitizes to various types of DNA damage including PARP-inhibition, G4-stabilization, UV, cisplatin, ATR inhibition, and Topoisomerase inhibition [84,110,134,135]. DDX11 facilitates DNA replication, fork restart, and the processing of G4 structures [84,125,130]. In response to DNA damage, Chl1/DDX11 promotes the formation of ssDNA as a suitable substrate for RPA loading, thereby contributing to efficient CHK1 activation, RAD51-dependent HR, and cohesin loading on stalled replication forks [58,85,135]. All functions of Chl1/DDX11 in DNA replication and repair depend on its helicase function [58,82,84,135]. In contrast, while the interaction of DDX11 with TIMELESS is important for efficient replication in response to HU and the processing of G4 structures [125,130], CPT and PARPi resistance are independent of this interaction [135]. In yeast, the interaction with Ctf4 is dispensable for HU resistance and RPA/cohesin loading at stalled forks [58,82]. Together, this suggests that additional mechanisms exist to recruit DDX11 to sites of DNA damage and the role of DDX11 in cohesion can be partially uncoupled from its role in the DNA damage response.

## 4. Establishment of Sister Chromatid Cohesion

### 4.1. SMC3 Acetyltransferases

In addition to the topological entrapment of sister chromatids, stable cohesion also requires the inhibition of the cohesin release activity of WAPL. In yeast, this is achieved by Eco1-dependent acetylation of the cohesin subunit SMC3 on K112/K113. The loss of *Eco1* or introduction of SMC3 acetylation-*null* mutations result in defects in cohesion and proliferation, which can be rescued by WAPL depletion [136,137,138,139,140,141]. Notably, *Eco1/Wpl1* double mutants have remaining cohesion defects, suggesting that Eco1 also possesses Wpl1-independent functions [136,138,142,143]. Human cells possess two *Eco1* orthologues: *ESCO1* and *ESCO2* [144]. The C-terminus is conserved, including the acetyltransferase domain, as well as its main substrate (SMC3 K105/K106 in human) [137]. ESCO1 and ESCO2 contain diversified, extended N-termini that are absent in yeast and bind different chromatin acceptors to allow different spatiotemporal control of acetyltransferase activity [45,144,145,146].

ESCO2 travels with the replisome to establish sister chromatid cohesion (Figure 2D) [145,147,148], functionally mimicking yeast Eco1 whose SMC3 acetylation depends on DNA replication [138,149]. Eco1/ESCO2 activity is limited to S phase by degradation via CUL4-DDB1-VPRBP and Anaphase Promoting Complex (APC/C) mediated ubiquitination (in yeast mediated by SCF^Cdc4^ (Skp1/Cul1/F-box)) [45,150,151,152]. A notable exception is DNA damage-induced cohesion establishment, resulting from the stabilization of Eco1 in G2 by Ddk inhibition and requiring acetylation of alternative cohesin residues [150,151,153].

In contrast to Eco1 and ESCO2, ESCO1 is not known to interact with the replication machinery. Instead, ESCO1 interacts with PDS5A/B, is able to acetylate SMC3 throughout interphase and is enriched at cohesin and CTCF binding sites [145,146,154,155]. This suggests that ESCO1 is not involved in DNA replication-coupled sister chromatid cohesion establishment. However, additive cohesion defects and lethality were observed upon ESCO1 depletion in ESCO2 deficient cells [97,145,147], and it has been proposed that ESCO1 functions in chromosome arm cohesion [44]. In addition, ESCO1 has functions in chromatin looping and transcription regulation [145,154,155].

### 4.2. Interactions of Eco1/ESCO2 with the Replisome

ESCO2 and Eco1 both interact with MCM via an N terminal interaction motif [44,45,148,156]. In addition, Eco1 interacts with PCNA via a PIP box and a possible second PCNA interaction motif [89,149,156]. ESCO2 also interacts with PCNA, which is facilitated by the conserved PIP box, as well as by two additional N-terminal PCNA binding domains [148,149]. While these PCNA and MCM interaction domains are highly conserved among vertebrates and all contribute to cohesion establishment, most of the ESCO2 N-terminus is predicted to be unstructured [44,148]. This may indicate that this region functions as a flexible linker, possibly extending up to 100 nm, allowing ESCO2 to make contact with multiple replisome components concomitantly [44,148]. Alternatively, these different interaction domains may facilitate ESCO2 positioning at multiple locations in the vicinity of the replication fork, which may contribute to acetylation of differently positioned cohesin complexes.

Preventing MCM loading by Cdc6 inhibition results in premature Eco1 degradation [152] and ESCO2 that lacks the MCM binding domain is destabilized and degraded by the CUL4-DDB1-VPRBP and APC/C complexes [45]. Both the PCNA and MCM binding domains contribute to SMC3 acetylation and Eco1 protein stabilization [149,156], although only the PCNA binding domains seem essential for growth [156]. Interestingly, the overexpression of an ESCO2 mutant that is defective in MCM-binding restores cohesion [44]. Furthermore, while the fusion of Eco1 to PCNA in an *Eco1*Δ background rescued the growth defect, fusion to MCM did not [89]. This suggests that the MCM interaction alone is insufficient to support sister chromatid cohesion. Therefore, the importance of the MCM interaction may be different from the direct positioning of ESCO2 for SMC3 acetylation at the replisome. Indeed, this interaction was reported to promote pre-RC recruitment of ESCO2 independent of replication fork activation [44,45,157]. Together, this suggests that the interaction with MCM functions in ESCO2 stabilization and pre-RC recruitment, and interactions with PCNA ensure the proper positioning for SMC3 acetylation behind the replication fork during active replication.

PCNA overexpression in temperature-sensitive *Eco1* mutants partially rescues viability and cohesion defects [59,158]. In line with this, the growth defects of a PCNA-binding mutant (Eco1^PIP^) can be rescued by fusing Eco1^PIP^ to Fen1 and (to a lesser extent) to Cdc9, which both function on the lagging strand in a late stage of DNA replication [89]. Together, this indicates that Eco1 functions in the wake of the DNA replication fork, where PCNA facilitates its ability to acetylate SMC3.

Furthermore, Eco1 was found to interact with the replication-fork-associated E3-ubiquitin ligase Rtt101-Mms1-Mms22, which contributes to SMC3-acetylation and cohesion independent of the Eco1–PCNA interaction [59]. The human homologue CUL4-DDB1-MMS22L also contributes to cohesion by binding to ESCO2 [159]. Rtt101-Mms1-Mms22 associates with the replisome through Ctf4 and is involved in histone transfer across the replication fork [160,161]. Similar to *Mms22* loss, mutation of the predominant ubiquitination targets to a non-ubiquitinatable form (H3K121R-K122R) similarly causes cohesion defects and Mms22 is epistatic with Ctf4 regarding cohesion defects [59].

In summary, Eco1/ESCO2 is recruited to replication forks by interactions with multiple replisome components, including MCM2-7, PCNA, and MMS22L, to efficiently establish sister chromatid cohesion.

### 4.3. SORORIN

In contrast to yeast, in vertebrates, SMC3 acetylation in itself is insufficient to stabilize cohesin onto chromatin but additionally requires SORORIN [15,162,163,164]. Acetylation of SMC3 allows the interaction of SORORIN with PDS5, thereby counteracting WAPL binding and cohesin release activity [15,165]. SORORIN is present during S/G2, but not in the G1 phase, when it is targeted for ubiquitin-dependent degradation by the APC/C-Cdh1 [163,164]. SORORIN does not contribute to stabilization of cohesin on chromatin in G1 and its association with cohesin occurs exclusively on replicated DNA [155,162]. The SORORIN-stabilized cohesin pool does not seem to contribute to intra-chromatid loops or topologically associating domain (TAD) formation but seems specifically required to stabilize sister chromatid cohesion [166]. The phosphorylation of SORORIN by the mitotic kinases Aurora B and CDK1 destabilizes the interaction of SORORIN with PDS5, rendering cohesin susceptible to WAPL release activity, which contributes to resolving cohesion in mitosis [15,167]. Thus, in vertebrates, SORORIN is essential to maintain sister chromatid cohesion by preventing the release of acetylated cohesin by inhibiting WAPL.

## 5. The Cohesin Complex and Associated Factors in the DNA Replication Stress Response

**Figure 3:**
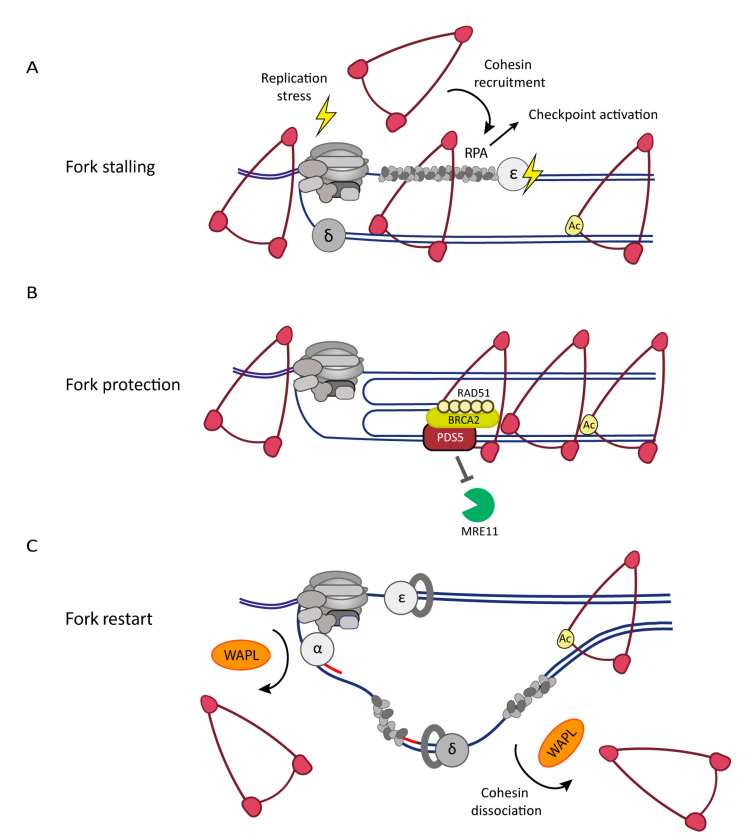
Cohesin dynamics in the DNA replication stress response: (**A**) Replication fork stalling can cause uncoupling of the polymerase from CMG, resulting in stretches of RPA-coated ssDNA that activate the replication checkpoint and are thought to promote cohesin recruitment. (**B**) The stalled fork undergoes extensive remodeling, often involving fork reversal to aid fork restart. Cohesin may be involved in protecting stalled replication forks and in facilitating template switching to repair DNA lesions. PDS5 contributes to the recruitment of BRCA2 and RAD51, which protect reversed forks against excessive nuclease processing. (**C**) Repair and restart depend on WAPL-mediated cohesin mobilization, possibly by increasing cohesin turnover at stalled forks. DNA replication is resumed while cohesin levels are decreased. Not all factors involved are shown in the figure for simplicity; for further details, see text.

DNA replication fork progression can be challenged by DNA lesions or shortage of metabolites for DNA synthesis, leading to transient replication fork progression defects. This so-called DNA replication stress can lead to stalling of DNA polymerases, resulting in the physical uncoupling of CMG from the polymerases. In addition, physical roadblocks impeding replicative helicase translocation can result in the nucleolytic processing of stalled forks. Both of these events result in the formation of stretches of ssDNA that are coated by the ssDNA binding protein replication protein A (RPA) (Figure 3A). Excess RPA-coated ssDNA is sensed by the DNA replication checkpoint involving the recruitment of the ATR kinase that activates CHK1 to coordinate the response to replication stress. In a process known as fork reversal or regression, stalled forks are often remodeled from standard three-way replication forks into four-way junctions by re-annealing of the parental strands and annealing of the newly synthesized strands, involving the activity of the RAD51 recombinase and SMARCAL1 translocase (Figure 3B). Fork regression is thought to have a physiological role in preventing more damage and promoting fork restart, but at the same time, reversed forks are vulnerable to excessive processing by nucleases, including the MRN complex (MRE11-RAD50-NBS1; MRX (Mre11-Rad50-Xrs2) in yeast) [168,169]. To prevent excessive nucleolytic activity, BRCA2 and RAD51 are recruited to protect reversed forks from degradation [169]. Nevertheless, prolonged stalling can result in fork breakage due to fork collapse or nucleolytic processing of replication intermediates. Thereby, replication stress contributes to genomic instability and is a hallmark of cancer [170].

### 5.1. Cohesin Dynamics and Replication Stress

Chromatin-bound cohesin complexes can form physical barriers for efficient DNA replication. Fork progression is hampered by both artificially tethering the SMC head domains, precluding ring opening [171], and by the depletion of WAPL and PDS5 [172,173]. Thereby, the removal of cohesin ahead of the replisome prevents cohesin from forming roadblocks for CMG progression to avert replication fork slow down, stalling, and the subsequent MRE11-dependent processing of stalled forks that would result in DSB formation [172,173]. Importantly, the overexpression of the oncogene c-MYC results in increased chromatin occupancy of cohesin and induces replication stress by increased replisome–cohesin collisions, suggesting that increased levels of chromatin-bound cohesin may be relevant in oncogenesis [174]. In addition to forming direct roadblocks for replisome progression, cohesin can trap torsional stress during DNA replication, which needs to be resolved by Topoisomerase II [175]. Together, this indicates that perturbed cohesin dynamics hamper replication fork progression.

Cohesin has been found to accumulate at stalled forks, possibly contributing to avert the damaging consequences of DNA replication stress (Figure 3A,B) [47,58]. This is promoted by Scc2 and the presence of stretches of ssDNA coated with RPA [58]. The combined activity of the MRX complex and Chl1 resects the stalled fork to form ssDNA coated with RPA as a template for cohesin loading [58]. Cohesin loading at stalled forks further depends on chromatin remodeling by the H3K4 methyltransferase Set1 and the histone acetyltransferase Gcn5 [58]. By keeping sister chromatids more tightly paired, cohesin could facilitate template switching to repair DNA lesions and promote efficient fork restart [47,87]. In line with such a model, increased expression of the cohesin subunit RAD21 mitigates replication stress in Ewing sarcoma cells [176]. In addition, cohesin may contribute to promote fork protection and homologous recombination (HR) by the PDS5-dependent recruitment of BRCA2 and RAD51 to stalled replication forks (Figure 3B) [173,177,178].

Interestingly, the inhibition of cohesin dissociation imparts cell viability in replication stress conditions in both budding yeast and higher organisms [173,179,180]. WAPL and PDS5 are both required for the repair of broken replication forks and efficient fork restart (Figure 3C) [172,179,180]. WAPL–PDS5 promotes the mobilization of cohesin and may thereby facilitate its accumulation at stalled forks to aid repair and restart [172,179]. Alternatively, WAPL may increase the turnover of cohesin during replication stress at sites of stalled or broken replication forks. This is reminiscent of DSB repair in post-replicative cells, which depends on cohesin dissociation for efficient end resection and DNA repair involving cohesin cleavage by localized SEPARASE activation [181,182,183]. While SEPARASE was also found to colocalize with MCM [46], at stalled replication forks, SEPARASE activity seems secondary to WAPL-dependent cohesin removal [180], suggesting that cohesin at stalled forks is removed before ESCO2/Eco1-dependent SMC3-acetylation, which would render cohesin resistant to WAPL activity. In yeast, Wpl-mediated cohesin mobilization at stalled forks depends on replication checkpoint dependent activation of Rsp5-Bul2 that ubiquitinates cohesin [179]. Surprisingly, the repair and restart of broken replication forks in WAPL-depleted cells can be rescued by reducing chromatin-bound cohesin levels [172,180], suggesting an excess of cohesin at stalled forks hampers DNA repair and fork restart. Notably, a recent analysis of the fork proteome following drug treatments suggests that cohesin components accumulate at DNA replication forks upon HU-induced stalling but not CPT-induced breakage [184]. Thus, the specific cohesin dynamics at replication forks are likely dictated by the kind of perturbation and the specific stage of the repair process.

### 5.2. DNA Replication Stress Affects Sister Chromatid Cohesion

The tight interconnection between DNA replication and sister chromatid cohesion suggests that defects in DNA replication can also impact sister chromatid cohesion. Multiple proteins have dual roles in sister chromatid cohesion and replication fork dynamics (see Box 1). Indeed, drugs causing replication stress result in sister chromatid cohesion defects [180,185,186]. Similarly, oncogene activation or loss of tumor suppressors that result in DNA replication stress can lead to cohesion loss, which can be rescued by nucleoside supplementation [180,187]. Interestingly, tumor cell lines, often experiencing DNA replication stress, exhibit significant cohesion defects that cannot be explained by direct mutations in cohesion genes [180,188,189]. DNA replication stress induced cohesion defects originate during DNA replication and lead to a decrease in chromatin-bound cohesin in G2 [180,185,187,190]. The link between replication stress and cohesion defects seems to be at least partially dependent on the cohesin removal activity of WAPL [180,186]. Possibly, the removal of cohesin at stalled replication forks comes at the cost of reduced sister chromatid cohesion. Interestingly, the ssDNA binding protein complex CTC1-STN1-TEN1 (CST) was recently shown to interact with cohesin and proposed to preserve functional cohesion in conditions of replication stress [191].

In summary, WAPL-dependent cohesin dissociation seems to play two roles at replication forks. First, cohesin removal from the front of the replisome prevents cohesin from forming roadblocks that hamper replisome progression [172,173]. Second, WAPL is involved in the response to DNA replication stress, by increasing cohesin mobility [179,180]. DNA replication stress can lead to loss of sister chromatid cohesion, in a process involving WAPL (Figure 3C) [180,186]. Further elucidation of the exact role of cohesin in the replication stress response will require a better characterization of the precise molecular contexts, such as type and severity of damage and the chosen repair pathway.

## Figures and Tables

**Table 1:** Key factors in sister chromatid cohesion and DNA replication.

	*Homo sapiens*	*S. pombe*	*S. cerevisiae*	Function
	SMC1A	Psm1	Smc1	Core cohesin subunits
	SMC3	Psm3	Smc3	
	RAD21	Rad21	Mcd1/Scc1	
	STAG1, STAG2	Psc3	Irr1/Scc3	Cohesin associated factors
	PDS5A, PDS5B	Pds5	Pds5	
	NIPBL	Mis4	Scc2	Cohesin loader complex
	MAU2	Ssl3	Scc4	
	WAPL	Wpl1	Rad61/Wpl1	Cohesin removal from chromatin
	ESPL1/SEPARASE	Cut1	Esp1	
	ESCO1, ESCO2	Eso1	Eco1/Ctf7	SMC3 acetylation
	SORORIN	-	-	WAPL antagonist
	CDC6	Cdc18	Cdc6	Pre-RC formation
	CDT1	Cdt1	Tah11/Cdt1	
	CDC45	Cdc45	Cdc45/Sld4	CMG (replicative helicase)
	MCM2-7	Mcm2-7	Mcm2-7	
	GINS1-4	Sld5-Psf1-Psf2-Psf3 (GINS)	Sld5-Psf1-Psf2-Psf3 (GINS)	
	PCNA	Pcn1	Pol30/Pcna	Polymerase sliding clamp
	POLA1-POLA2-PRIM1-PRIM2	Pol1-Spb70-Spp1-Spp2	Pol1-Pol12-Pri1-Pri2	Polymerase α-Primase complex
	POLD1-4	Cdc6-Cdc1-Cdc27-Cdm1	Pol3-Pol31-Pol32	Polymerase δ complex
	POLE-POLE2-POLE3-POLE4	Cdc20-Dpb2-Dpb3-Dpb4	Pol2-Dpb2-Dpb3-Dpb4	Polymerase ε complex
	FEN1 DNA2	Rad2 Dna2	Rad27 Dna2	Okazaki fragment processing
	LIG1	Cdc17	Cdc9	
Epistasis group 1	AND-1/WHDH1	Mcl1	Ctf4	Mediating interactions at the replication fork
	DDX11	Chl1	Chl1	DNA helicase
	TIMELESS	Swi1	Tof1	Fork Protection Complex (FPC)
	TIPIN	Swi3	Csm3	
Epistasis group 2	CLASPIN	Mrc1	Mrc1	
	CHTF18	Ctf18	Ctf18	PCNA loader on leading strand (with Rfc2-5)
	CHTF8	Ctf8	Ctf8	
	DSCC1	Dcc1	Dcc1	
	RFC1	Rfc1	Rfc1/Cdc44	PCNA loader on lagging strand (with Rfc2-5)
	ATAD5	Elg1	Elg1	PCNA unloader (with Rfc2-5)
